# Plasma lipidomic analysis shows a disease progression signature in *mdx* mice

**DOI:** 10.1038/s41598-021-92406-6

**Published:** 2021-06-21

**Authors:** Roula Tsonaka, Alexandre Seyer, Annemieke Aartsma-Rus, Pietro Spitali

**Affiliations:** 1 Biomedical Data Sciences, Leiden University Medical Center, Einthovenweg 20, 2333 ZC Leiden, The Netherlands; 2 MedDay Pharma, 24 Rue de la Pépinière, 75008 Paris, France; 3 Department of Human Genetics, Leiden University Medical Center, Einthovenweg 20, 2333 ZC Leiden, The Netherlands

**Keywords:** Genetics, Neurology, Metabolomics

## Abstract

Duchenne muscular dystrophy (DMD) is a rare genetic disorder affecting paediatric patients. The disease course is characterized by loss of muscle mass, which is rapidly substituted by fibrotic and adipose tissue. Clinical and preclinical models have clarified the processes leading to muscle damage and myofiber degeneration. Analysis of the fat component is however emerging as more evidence shows how muscle fat fraction is associated with patient performance and prognosis. In this article we aimed to study whether alterations exist in the composition of lipids in plasma samples obtained from mouse models. Analysis of plasma samples was performed in 4 mouse models of DMD and wild-type mice by LC–MS. Longitudinal samplings of individual mice covering an observational period of 7 months were obtained to cover the different phases of the disease. We report clear elevation of glycerolipids and glycerophospholipids families in dystrophic mice compared to healthy mice. Triacylglycerols were the strongest contributors to the signatures in mice. Annotation of individual lipids confirmed the elevation of lipids belonging to these families as strongest discriminants between healthy and dystrophic mice. A few sphingolipids (such as ganglioside GM2, sphingomyelin and ceramide), sterol lipids (such as cholesteryl oleate and cholesteryl arachidonate) and a fatty acyl (stearic acid) were also found to be affected in dystrophic mice. Analysis of serum and plasma samples show how several lipids are affected in dystrophic mice affected by muscular dystrophy. This study sets the basis to further investigations to understand how the lipid signature relates to the disease biology and muscle performance.

## Introduction

Duchenne muscular dystrophy (DMD) is a rare neuromuscular disorder affecting 1 in 5000 male births^1^. The disease is caused by mutations in the dystrophin encoding *DMD* gene causing lack of dystrophin^2^. Patients with DMD show delayed motor development, muscle weakness, wheelchair dependency and premature death^3^. The progressive nature of the disease is explained by the gradual substitution of muscle mass with fibrotic and adipose tissue^4^. Although the accumulation of fat in skeletal muscles differs across muscle groups and across patients, the fat deposition pattern across muscle groups is typical in DMD patients and it has been recently shown to have both diagnostic^5^ and prognostic capacity^6^. Indeed, muscle fat fraction has been included as primary or secondary outcome measure in interventional clinical trials (e.g. NCT01388764 and NCT02851797) and natural history studies (NCT01451281). While fat infiltration is related to reduced force generation during muscle contraction, ectopic fat has also been linked to diabetes risk in the general population as well as in adolescents^7, 8^.

In the recent past it has been shown that DMD boys show signs of insulin resistance^9^ and metabolic syndrome^10^, consistent with an increase in fat mass compared to healthy boys^11, 12^. Evidence from proteomic studies supported the potential association with metabolic syndrome as an increase in metabolic syndrome markers such as leptin in both corticosteroid treated^13, 14^ and untreated patients^10^ was reported. In line with the fat deposition in muscle, increased serum concentration of triglycerides, phospholipids, free cholesterol, cholesterol esters and total cholesterol were observed in DMD patients compared to age-matched controls^10, 15^. Further reports showed how DMD patients have high levels of adiponectin in serum compared to healthy controls and that the concentrations increase over time^16, 17^. Adiponectin is mainly secreted by adipose tissue and it is considered to mirror the increase in fat accumulation in muscle^17, 18^. While this evidence is not sufficient to diagnose DMD patients with metabolic syndrome, there is undisputed evidence of fat accumulation in DMD patients’ muscles and substantial support of a local and circulating signature of adipose tissue in DMD.

A few reports investigated the lipid content in DMD skeletal muscle. An increase in sphingomyelin, cholesterol, triglycerides and reduced acylation of phosphatidylcholine with linoleic acid was consistently reported in previous studies in muscle biopsies^19–22^. Specifically, the colocalization of cholesterol, sphingomyelin and phosphatidylcholine in highly degenerative areas suggests differential lipid regulation in damaged regions, but an increase in the relative abundance of mono-unsaturated and saturated fatty acids of phosphatidylcholine suggests reduced membrane flexibility of muscle fibers in DMD^23^.

While the analysis of lipids in muscle biopsies has provided some insight in the lipid composition in dystrophic muscle, longitudinal characterization of the changes upon disease progression is missing due to the impossibility to perform repeated biopsies over time for research purposes. Given that associations in the composition of lipids between serum and muscle have been described^24^, we focused our investigation on blood samples, which offer the possibility to obtain repeated samplings and observe changes during follow-up visits. In this study we collected evidence in mouse models to understand whether a lipidomic signature could support future studies in humans. We used the *mdx* mouse model, which is the most widely used murine model for DMD, carrying a nonsense mutation in exon 23. Plasma samples were obtained for healthy and dystrophic mice over a period of 7 months at 5 phased time points corresponding to different phases of the disease. We identified a strong signature of different lipid classes across the whole observational time, showing that a signature composed of different lipids is present beyond the early phase of the disease where heavy muscle degeneration and regeneration is occurring. The signature was further investigated in additional mouse models with a different genetic background carrying 2, 1 or no functional copies of the *Utrn* gene, encoding the dystrophin paralog utrophin. As utrophin can partly compensate for lack of dystrophin in mice, *Utrn* up-regulation has been proposed as therapeutic strategy for DMD; the mice included in this study have been previously shown to have subtle phenotypic differences related to the copy number of functional *Utrn*^25, 26^. The obtained data support the investigation of circulating lipids in larger animal models as well as in patients affected by Duchenne muscular dystrophy.

## Materials and methods

### Mice

All mice involved in the experiments were males and they were housed in individually ventilated cages. Five groups of mice were included in the experiment. Heathy WT and *mdx* mice (lacking dystrophin due to a nonsense mutation in exon 23) shared the same genetic background (C57BL/10ScSn). We also included *mdx* mice with different copy number of functional utrophin (2, 1 and 0 functional copies). These mice have a mixed genetic background (not C57BL/10ScSn as WT and *mdx*). Both *mdx* mice and *mdx utrn*^+/+^ have 2 functional utrophin copies but they have a different genetic background. Mice had ad libitum access to chow and free access to water. Blood was obtained from the tail vein at 6, 12, 18 and 24 weeks of age and from the eye at week 30. Before sampling, mice did not have access to food for 4–6 h; however, they had free access to water. Anaesthesia with isoflurane was used before sampling. A solution of lidocaine and adrenaline was applied locally after blood withdrawal and before mice were allowed to wake up. Blood samples were obtained in heparin lithium tubes. At week 30, mice were sacrificed by cervical dislocation. The experiment consisted of 5 different groups of mice including WT, *mdx*, *mdx* mice carrying 0, 1 or 2 functional utrophin copies. Each group consisted of 5 mice. One *mdx utrn*^+*/−*^ mouse was humanely sacrificed at 8 weeks of age according to the protocol, due to weight loss and no recovery; therefore an extra mouse was added to this group to compensate for the early drop out. For the double knock-out mice only samples at 6 weeks of age were collected; double knock out mice were humanely sacrificed at 6 weeks of age before the onset of severe pathology. Samplings and analysis were not successful at all time points, therefore some data were missing. Overall, we obtained single measurements for 9 mice, 2 longitudinal data points for 2 mice, 3 repeated measurements for 3 mice, 4 and 5 longitudinal observations for 8 and 4 mice, respectively. The experiment was evaluated and approved by the local animal welfare committee (Dierexperimentencommissie Academisch Ziekenhuis Leiden, based at the Leiden University Medical Center) under DEC number 13154. All the animal experiments were carried out in compliance with ARRIVE guidelines. All methods were carried out in accordance with relevant guidelines and regulations.

### Data acquisition

Murine plasma samples were obtained by centrifuging blood at 18,000 g for 5 min at 4 °C. The supernatant was frozen at −80 °C. Serum samples were extracted using an adaption of methods previously described^27^. Briefly, 50 µL of each plasma samples were was added to 245 µL CHCl3/MeOH 1:1 (v/v) and 5 µL of a mix of internal standards. After a 2 h extraction at 4 °C, 37 µL of water was added, samples were mixed and centrifuged. The upper phase (aqueous phase) and the lower lipid-rich phase (organic phase) were pooled and dried under nitrogen. Samples were then reconstituted with one 100-fold dilutions of initial volume (50 µl) of MeOH/IPA/H2O 65:35:5 (v/v/v). Serum total lipid extracts were separated on a Transcend 1250 liquid chromatographic (LC) system (ThermoFisher Scientific, Les Ulis, France) using a kinetex C8 2.6 µm 2.1 × 150 mm column (Phenomenex, Sydney, NSW, Australia). The LC system was coupled to a Q-Exactive mass spectrometer (Thermo Scientific, San Jose, CA). Quality control samples were included all along the analytical sequence, and successive dilutions along with blank series at the beginning. Analysis in positive and negative ionization modes were done in two separates runs. Data were processed as previously described^27^, i.e. XCMS was used for features detection^28–30^, and the dataset were filtered according the coefficient of correlation, the coefficients of variation and the ratio of chromatographic area of biological to blank samples^31^. Annotation of features was done based on the exact m/z ratio, retention time range and relative isotopic ratio (RIA) thanks to an in silico lipid database. Finally, formal identification of compounds of interest was obtained thanks to targeted tandem mass spectrometry experiments in higher energy collision-induced dissociation (HCD) conditions performed on QC samples.

### Statistics

Data exploration was performed including all time points and strains. Data normalization was performed by dividing concentration by the standard deviation. Principal component analysis (PCA) including all unique lipids was performed, followed by hierarchical clustering (complete linkage) of the Pearson correlation matrix to identify correlating lipids. Data exploration was followed by formal data analysis, which included first the analysis at the lipid family level, followed by analysis of the individual lipids. Initial analysis included WT and *mdx* mice only. The identified signatures were then explored in *mdx* mice carrying 0, 1 and 2 functional copies of utrophin. Lipids families and subclass data were obtained by summing the concentration of each individual lipid belonging to the families and subclass, respectively. Each lipid family was analysed using linear mixed models to capture the within mice correlations due to the repeated lipid measurements over time^32^. Time in weeks (categorical), genotype (WT, *mdx*) and the interaction between time and genotype were included as fixed effects. We modelled the correlation within mice over time using random intercepts and random slopes terms. The null hypothesis was that differences did not exist between groups at baseline (genotype main effect) and over time (interaction between time and group). As main effects and interaction were tested jointly, a global *P* value (*P.global*) is reported. The likelihood ratio test was used and *P*-values below 0.05 were considered significant. Differences between *mdx* and WT mice in the progression (interaction time and genotype) and at each time point were tested with Wald tests at each time point. For certain samples and time points the analysis was not possible due to the limited volume of plasma available resulting in missing data. Data for *mdx* mice were missing for the 18 week time-point as the obtained plasma volume was not sufficient to perform the analysis. For lipid families showing significant associations, similar models were performed to assess differences between *mdx* utrn^+/+^, *mdx* utrn^+/−^ and *mdx* utrn^−/−^ mice. No multiple testing corrections were applied for the analysis of lipid families as only 5 families were tested. For individual lipids a similar model was used, however we were forced to exclude *mdx* utrn^−/−^ mice from the analysis, as models did not converge when these mice were included. This complication is due to the fact that only the first time point was available for these mice, limiting the possibility to assess whether differences exist in the disease progression trajectories of these mice. The obtained *P*-values were corrected for multiple testing by Benjamini–Hochberg false discovery rate (FDR)^33^ and a threshold of 0.05 was considered significant. All analyses and visualizations were performed in R using packages *corrplot*, *nlme*, *splines*, *lattice*, *aod*, *ggplot2*, *lm*, *prcom*, *circlize, dplyr* and *tidyr*.

## Results

**Figure 1:**
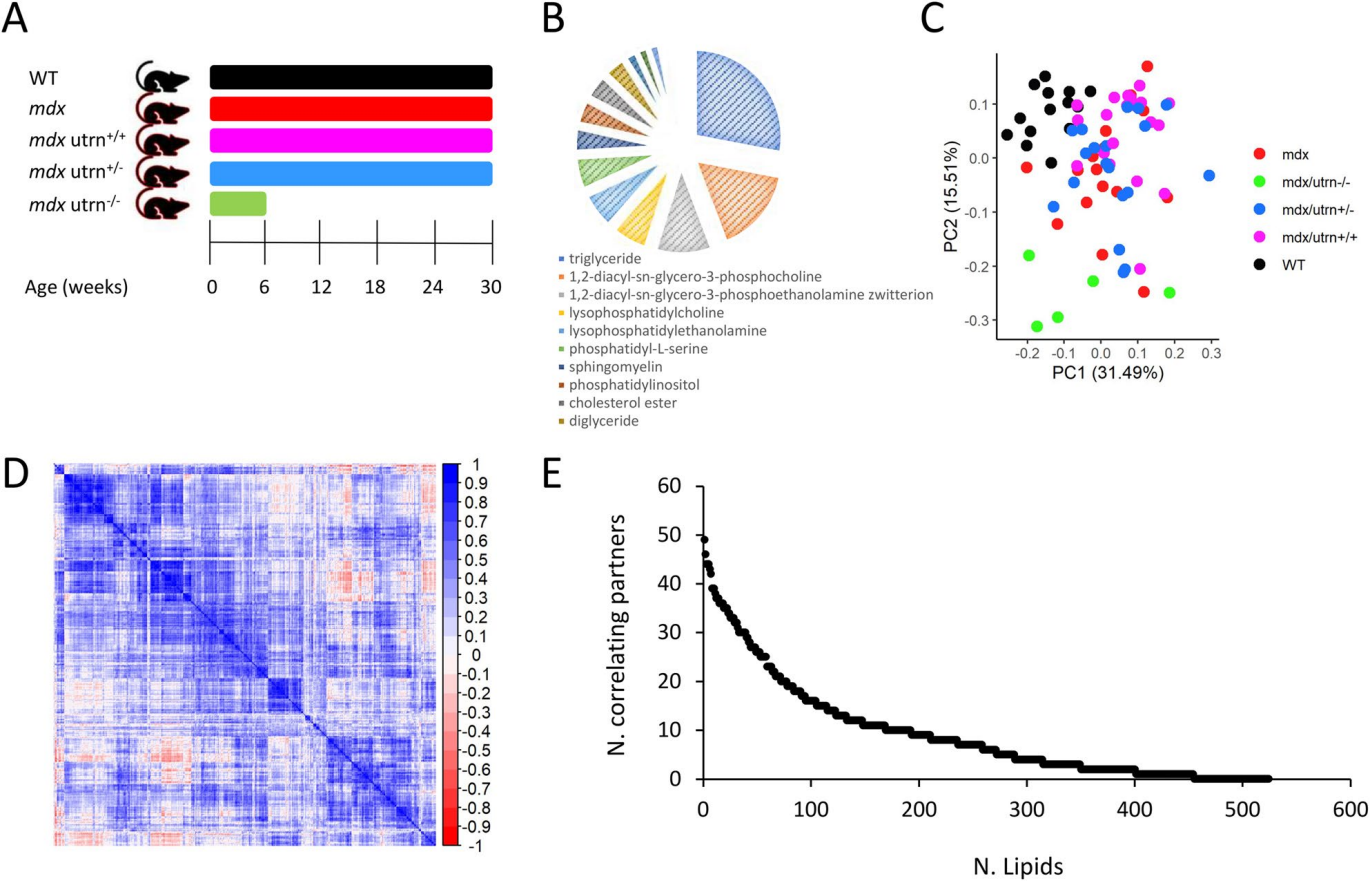
Study overview. (**A**) Graph showing the study duration and age at sampling for WT, *mdx* and *mdx* mice with 2, 1 and no functional utrophin alleles. Samples were obtained at 6, 12, 18, 24 and 30 weeks of age. (**B**) Pie chart showing the lipid subclasses identified in mice plasma according to ChEBI names. We included in this chart subclasses that had more than 6 lipids in the dataset to improve readability. (**C**) Scatter plot showing the first 2 principal components for all analyzed samples. Colors indicate mice with different genotypes. WT mice (black) cluster at top left corner, while double knock out mice (green) are located at the bottom of the graph. The other 3 genotypes show large overlap. (**D**) Heatmap showing the hierarchical clustering of the correlation matrix for all lipids. Blocks with moderate correlations are visible. (**E**) Scatter plot showing for each detected lipid (x-axis) the number correlated lipids (Pearson absolute R value > 0.8).

In DMD, muscle mass is replaced by fat and fibrotic tissue. The percentage of muscle substituted with fat has been shown to accumulate over time and to predict disease milestones^6^. In this work we sought to identify whether lipids in circulation are associated with disease progression in dystrophic mice. To study the association of lipids and disease progression we designed a longitudinal pre-clinical study in dystrophic and healthy mice, where plasma samples were collected every 6 weeks starting at 6 weeks of age up to 30 weeks of age (Fig. 1a). A total of 524 lipids were analysed comprising 5 lipid families (fatty acyls, glycerolipids, glycerophospholipid, sphingolipids and sterol lipids). Figure 1b shows a schematic representation of the sample composition in lipid sub-classes. Comparison of WT and *mdx* mice was performed with the intention to identify lipids associated with lack of dystrophin and lipids showing different progression over time. Sample volumes did not allow analysis of *mdx* samples at 18 weeks of age, therefore only 4 time points were available for *mdx* mice. Another 3 *mdx* mouse models carrying 2 (*mdx utrn*^+/+^), 1 (*mdx utrn*^+/−^) or no (*mdx utrn*^−/−^) functional alleles coding for the dystrophin paralog utrophin were included, as these mice show somewhat different progression due to dose dependent compensation by functional utrophin alleles. For *mdx utrn*^−/−^ mice, only samples at 6 weeks were collected, as these mice are too severely affected to allow longer observational time.

Initial data exploration by principal component analysis (PCA) showed that samples distributed in the space forming 3 main clusters with WT mice, *mdx utrn*^−/−^ mice, and the other *mdx* mice with at least 1 functional utrophin allele (Fig. 1c). No clear separation was visible in the third cluster with *mdx*, *mdx utrn*^+/+^ and *mdx utrn*^+/−^ mice. To inspect whether correlations exist across lipids, we plotted the clustered correlation matrix for all lipids, showing that moderate correlation was present in the data (Fig. 1d). We explored the strength of the correlation across lipids by considering all correlating pairs with an absolute Pearson correlation value above 0.8. A total of 454 lipids had at least 1 correlating partner beyond the 0.8 threshold. Although a total of 77 lipids had 20 or more correlating partners, the correlation was distributed across the dataset with multiple relatively small correlating blocks (Fig. 1d). The distribution of the number of correlating partners for each lipid shows how only a small fraction of lipids has more than 40 correlating partners above the selected 0.8 threshold (Fig. 1e).

**Figure 2:**
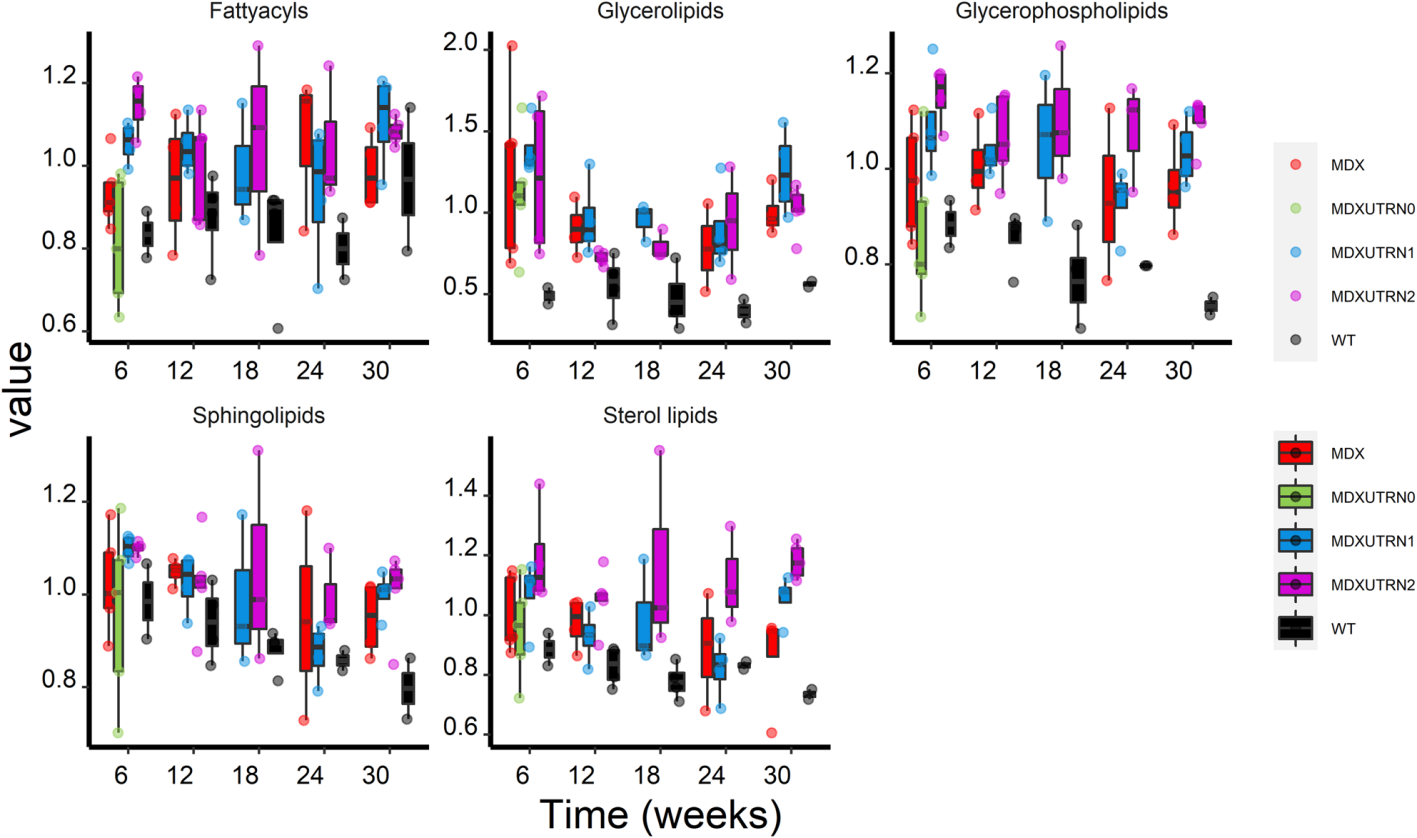
Analysis of lipid families over time in WT, *mdx* and *mdx* utrophin mice. Box plots showing the progression of each lipid family over the 30 weeks observational time. The cumulative levels of each lipid family (sum of all lipids belonging to the class) are plotted on the y axis for fatty acyls, glycerolipids, glycerophospholipids, sphingolipids and sterol lipids. *Mdx* mice are plotted in red, WT in black; *mdx* mice with 0, 1 and 2 functional utrophin alleles are plotted in green, blue and pink, respectively.

Analysis of lipids at the lipid family level allowed comparison of fatty acyls, glycerolipids, glycerophospholipids, sphingolipids and sterol lipids over the study duration. A significant effect of group and interaction between group and time was observed between *mdx* and WT mice for glycerolipids and glycerophospholipids (*P* < 0.05 for both lipid families). To assess whether differences existed at specific time points or whether they were conserved throughout the whole time on study, Wald tests for the individual time points were performed showing that glycerolipids were significantly elevated at all time points, while glycerophospholipids showed significant elevation only at week 12 and 30 (Fig. 2 and Table 1).

**Table 1:** Table showing the comparison of the 5 lipid families between WT and mdx mice.

	Week6	Week 12	Week24	Week30	*P*.*global*
Fatty acyls					0.1877
Glycerolipids	** < 0.0001**	** < 0.0001**	** < 0.0001**	** < 0.0001**	**0.0283**
Glycerophospholipid	0.306	**0.012**	0.190	**0.008**	**0.0396**
Sphingolipids					0.2970
Sterol lipids					0.1002

The *P.global* reports whether any significant association was detected for each lipid family between *mdx* and WT mice at baseline and over time. In case of *P.global* < 0.05 weekly comparisons across mice groups were performed. Significant associations are shown in bold. Comparison of WT and *mdx* mice was not possible at 18 weeks due to the lack of *mdx* samples at this specific time point.

To assess whether the identified signature was associated with disease severity, we tested whether the concentration of glycerolipids and glycerophospholipids was associated with the number of functional utrophin alleles in *mdx* mice carrying 0, 1 or 2 functional utrophin alleles. Significant differences across groups were found only for glycerophospholipids (*P* < 0.01); differences were found in 6 weeks old mice, where glycerophospholipids were lower in double knock out mice compared to *mdx* mice with 1 and 2 functional utrophin copies (*P* < 10^−3^ and *P* < 10^−4^, respectively), and at 24 weeks between *mdx utrn*^+/+^ and *mdx utrn*^+/−^ mice (*P* < 0.05) (Fig. 2).

**Figure 3:**
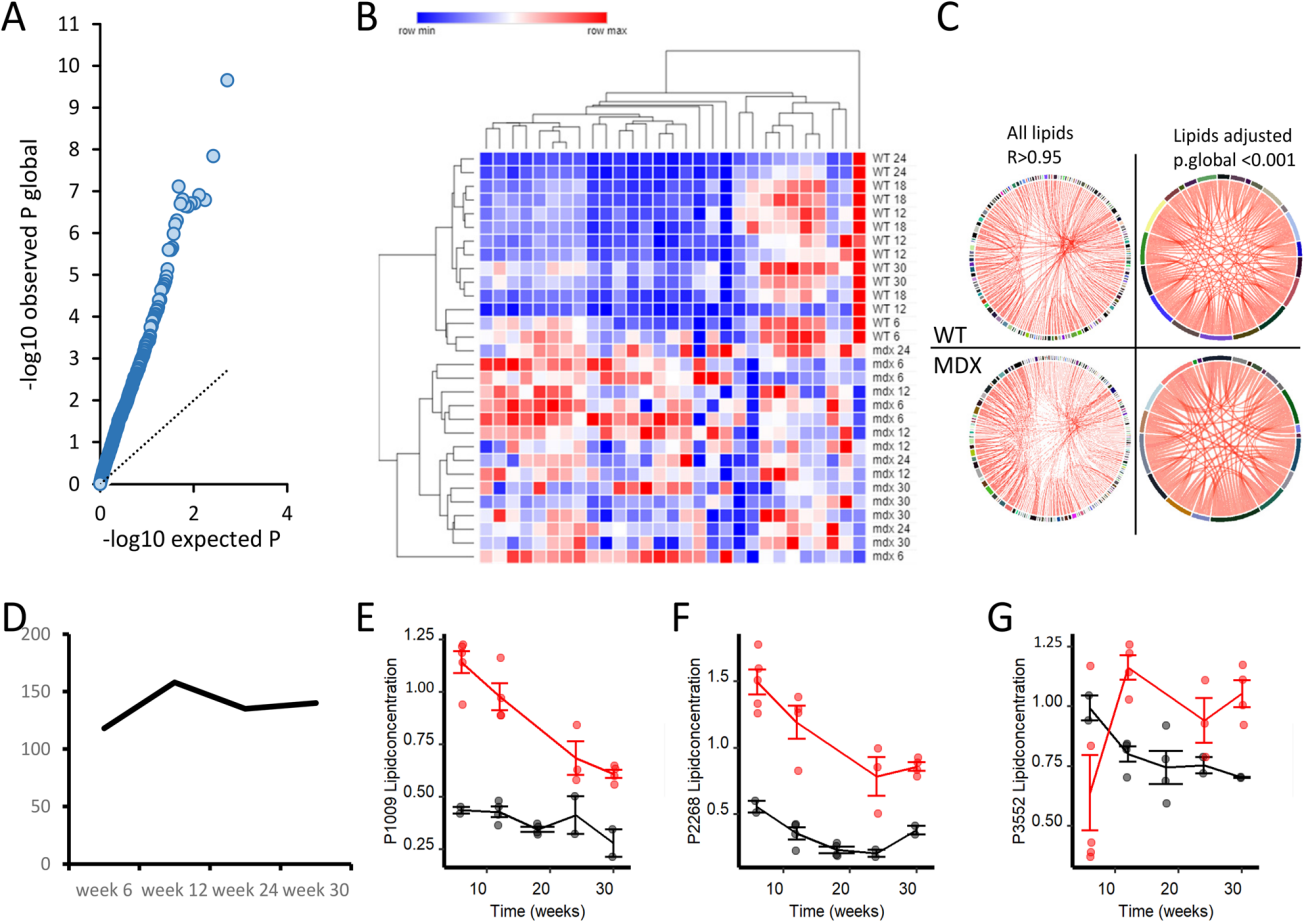
Analysis of individual lipid trajectories in *mdx* and WT mice. (**A**) Scatter plot showing the enrichment in low *P* values in the observed distribution compared to the expected one. (B) Heatmap showing the abundance of 29 lipids with adjusted *P.global* < 0.001. Hierarchical clustering shows complete separation of dystrophic and WT animals. (**C**) Circular plots showing the correlation across all lipids and the 29 selected lipids in both WT and *mdx* mice. Despite the difference in abundance, the correlation across the lipids was maintained. (**D**) Plot showing the number of lipids significantly dysregulated at each time point in *mdx* mice. At 12 weeks of age the highest number differences was recorded. (**E**–**G**) Line plots showing the differences for 3 specific lipids at all time points.

Given that assessment at the lipid family level could hide differences at the unique lipid level, we tested whether differences were present at the individual lipid level. We modelled the individual mice trajectories to test whether differences exist between *mdx* and WT groups at any time point and to test whether changes in the progression over time were different between the groups. A total of 160 lipids were significant after multiple testing correction (Table S1), showing that in at least one instant in time there was difference between healthy and dystrophic mice (Fig. 3a). Hierarchical clustering based on 29 lipids with adjusted *P.global* < 0.001 showed complete clustering of WT and *mdx* mice, with the later points in time being further separated from the early ones (Fig. 3b). Correlation analysis across these 29 lipids showed that the correlation across lipids was preserved in *mdx* mice for the majority of the lipids despite the differences in plasma concentration (Fig. 3c). We proceeded to identify the moment in time where significant differences occurred for each of the 160 lipids with adjusted *P.global* < 0.05. The number of lipids able to separate *mdx* and WT mice was roughly the same for all time points with majority of lipids showing increased levels in *mdx* mice compared to WT (Fig. 3d). Figure 3e–g and Figure S1 show examples of lipids levels in *mdx* mice plasma compared to WT mice. In line with the analysis at the family level, lipids belonging to glycerolipid and glycerophoispholipid families were the largest contributors at the unique lipid level with 145 out of the 160 belonging to these families (Table 2). A total of 50 lipids were significant at all time points with 38 triacylglycerols showing *P* < 0.0001 at all timepoints (Table S1). We further tested whether any of the 160 lipids identified in the comparison of WT and *mdx* mice was associated with the number of functional utrophin alleles in *mdx* utrophin mice. No significant associations were identified.

**Table 2:** Table showing the distribution of the 160 significant lipids through lipid family and subclass.

Lipid class and family	Count
**Fatty acyls**	**1**
Free fatty acids	1
**Glycerolipids**	**77**
Diacylglycerols	4
Triacylglycerols	73
**Glycerophospholipids**	**68**
Glycerophosphocholines	24
Glycerophosphoethanolamines	16
Glycerophosphoglycerols	3
Glycerophosphoinositols	4
Glycerophosphoserines	1
Lyso-Glycerophosphocholines	12
Lyso-Glycerophosphoethanolamines	7
Lyso-Glycerophosphoglycerols	1
**Sphingolipids**	**10**
Ceramides	1
Gangliosides	4
Sphingomyelins	3
Sulfoglycosphingolipids	2
**Sterol lipids**	**4**
Cholesteryl esters	4
**Grand Total**	**160**

The number of lipids per family and subclass is provided.

## Discussion

Muscular dystrophies are highly disabling disorders characterized by progressive loss of muscle function. A common feature among many muscular dystrophies is the substitution of muscle mass with fibrotic and adipose tissue. While the process of muscle substitution by fat is not well understood, methods able to quantify muscle fat fraction are used as tools to predict disease progression as well as outcome measures in clinical trials^6^. Duchenne muscular dystrophy is one of the most severe and common forms, where fat accumulation happens relatively quickly, although at different levels per muscle group^34^. Analysis of lipids has been performed in muscle biopsies obtained in small groups of patients^21^, showing how the lipid profile is affected in degenerating and regenerating muscle fibers. Only a few studies explored whether a lipid signature exists in more accessible samples such as blood^15, 35, 36^. Given that lipidomics profiles have been shown to be comparable across species, especially between human and mouse^37^, we sought to study lipid composition in plasma and serum samples of dystrophic mice to understand whether a lipid signature is associated with disease progression. Lipidomic analysis of plasma samples of WT and *mdx* mice was performed on longitudinal samples obtained in an observational study of 7 months. Analysis at the lipid family level allowed the identification of differences for glycerolipids and to a lower extent for glycerophospholipids. Glycerolipids showed consistent elevation at all time points. Analysis at the unique lipid levels confirmed the analysis at the family levels; indeed, of the 160 lipids significantly altered in *mdx* mice, 145 belonged to the glycerolipid and glycerophospholipid families. Triacylglycerols largely contributed to the glycerolipids signature, while diacylglycerols were less represented. This finding is in line with previous observations in *mdx* mice, where triglycerides levels were reported to be as high as 140 mg/dl after over-night fasting^38^, when the normal range of triglycerides levels in WT mice were reported in other studies to be between 30 and 50 mg/dl^39–41^. Of note a different study did not observe a significant difference between *mdx* and WT, perhaps due to the fact that mice were not fasted when the samples were obtained or perhaps due to the high levels present in WT animals in this study (reported in 0.9 mmol/l corresponding to ~ 80 mg/dl)^42^.

Elevation of triglycerides was previously reported in patients in two previously publications. A study performed in cohort of DMD patients followed up in India, reported values of 41 DMD patients (1.27 mmol/l) to be higher than healthy age matched controls (0.40 mmol/l)^15^. While elevated triglycerides levels in DMD patients corroborates our findings, the triglycerides levels of healthy control reported in this study were particularly low compared to the normal range recently reported by the American College of Cardiology/American Heart Association for children^43^ and they were in line with control paediatric population reported by the National Health and Nutrition Examination Survey (< 150 mg/dl)^44^. Furthermore triglycerides levels reported in the study were also matching the regional reference values as they were reported for the Indian paediatric population (< 1.7 mmol/l)^45^. Another recent paper reported elevation of triglycerides in young BMD and DMD patients compared to the normal range reported by the American College of Cardiology^36^. The authors show compelling evidence that lack or reduced levels of dystrophin may lead to dyslipidemia in humans and dogs. While there is a clear effect on triglycerides, it has also to be noted that the majority of patients show borderline elevation according to the definition of the American College of Cardiology (also used by the authors) and that samples in the study were obtained in non-fasted patients. In our view it is therefore possible that the elevation observed in mice could be reflected in patients as well but that the evidence collected so far is confounded by food intake as it is known that food intake affects triglycerides levels^46^. The difference in the effect sizes observed between dystrophic mice and patients could be due to the different composition of muscle fibres in two species. Murine muscles are composed of more fast twitch fibres compared to human muscle fibres^47^. Fast twitch fibres are metabolically more active and richer in glycogen, while slow twitch fibres are more dense in mitochondria and they have the capacity to accommodate higher concentration of lipids such as triglycerides^47^. Murine fast muscles may be therefore less able to absorb triglycerides compared to humans, leading to the detection of elevated triglycerides levels in blood; dystrophic patients instead can accommodate more lipids in their muscle fibres (as shown by MRI with increase fat fraction^6, 34^), corresponding to borderline concentration of triglycerides in circulation. This could create the conditions for a more efficient substitution of muscle with fat, which is typical in DMD patients and almost entirely absent in *mdx* mice. While questions remain on how fat is deposited in Duchenne patients’ muscle driving the substitution of muscle mass with adipose tissue, the metabolism of triglycerides seems an important of point in this mechanism as it has been recently shown that *mdx* mice lacking the *ApoE* gene show increased fat deposition in muscle and resemble more closely the histological features observed in patients^42^.

The association of the observed lipids signature with disease progression relates to the histological findings described in literature in dystrophic mice. Indeed, while at 6 to 12 weeks of age *mdx* mice show large areas of inflammation and ongoing muscle regeneration, later time points such as 24 to 30 weeks of age show less (clustered) inflammation in combination with increased fibrosis^48^ and switch towards slower twitch fibers^49^. Our data show that despite differences in lipid concentration remain significant, the large fold changes observed at the early time points are somewhat reduced towards the later time points, possibly due to the extravasation of plasma lipids into tissues due to vascular dysfunction as previously hypothesized^42^, and to the increase capacity of muscle to store lipids thanks to the increase in slow twitch fibers content. The extravasation of lipids into tissue could therefore be visualized as decreased plasma lipid signature in combination with an increase presence of adipose tissue in muscle, as it is observed for example in the diaphragm muscle at later time points^50^.

In conclusion, our study has identified a strong lipidomic signature in dystrophic mice related to disease progression given the associations of plasma lipids and especially triglycerides with age in dystrophic mice. The evidence presented in this report supports the investigation of lipid metabolism in patients to understand if and how lipids and specifically triglycerides contribute to fat deposition in muscle tissue and in different muscle fibers.

## Supplementary Information

**Figure S1**. Box plots showing differences in plasma lipids levels between mdx and WT mice for all lipids with adjusted P.global <0.01.

**Table S1**. List of 160 lipids significantly altered in *mdx* mice compared to WT mice in at least one instant in time (FDR<0.05). Global unadjusted and adjusted P values are reported as well as time point specific Wald test results.
