# Supplementary figures and images for: Plasma lipidomic analysis shows a disease progression signature in mdx mice
Source: Sci Rep. 2021 Jun 21;11:12993. doi: 10.1038/s41598-021-92406-6 (PMC8217252; doi:10.1038/s41598-021-92406-6)

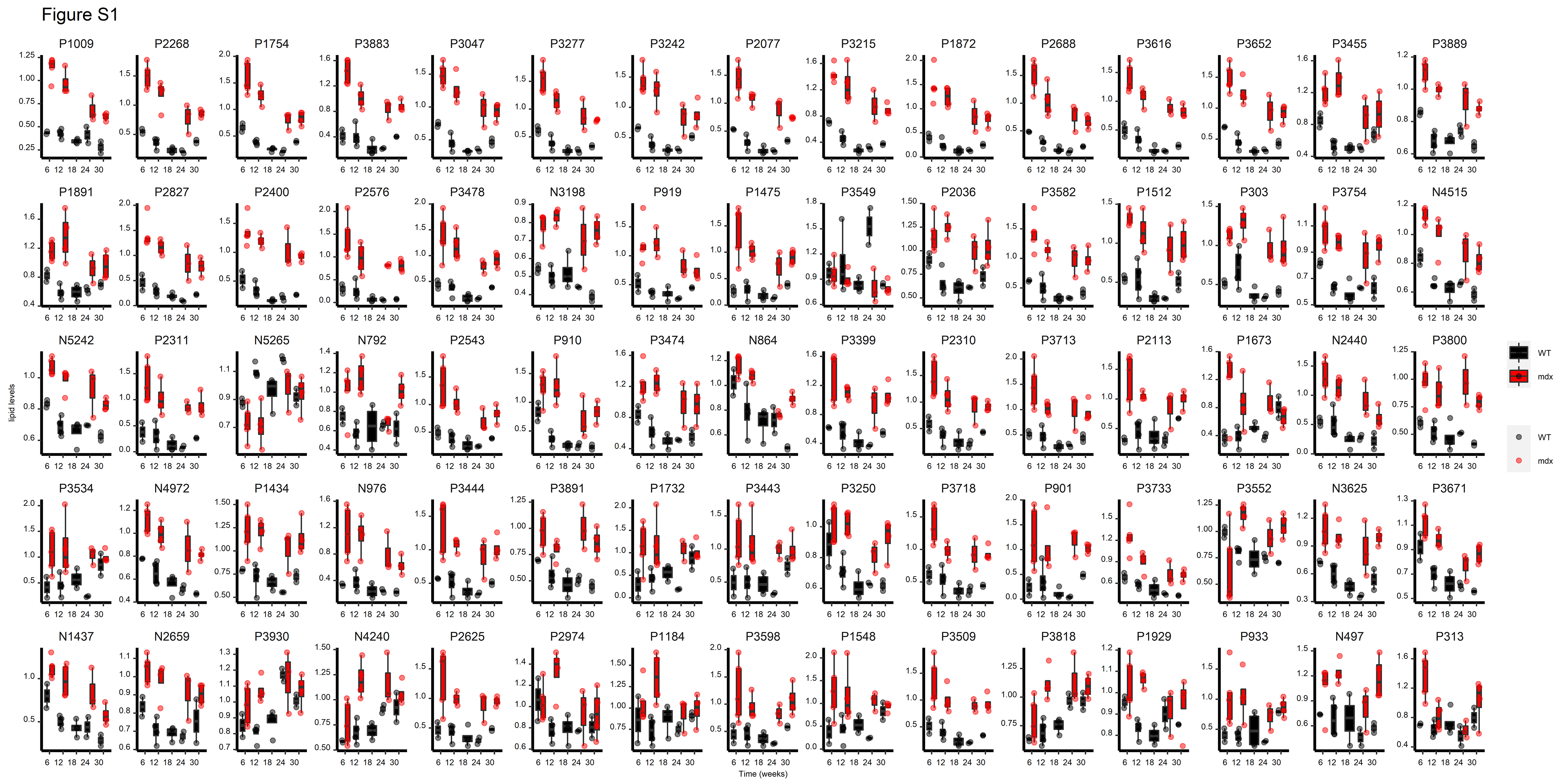

Supplement: Supplementary file 1 — Figure S1. Box plots showing differences in plasma lipids levels between mdx and WT mice for all lipids with adjusted P.global <0.01. [file 41598_2021_92406_MOESM1_ESM.tiff]
